# Factor associated with experience of modern contraceptive use before pregnancy among women who gave birth in Kersa HDSS, Ethiopia

**DOI:** 10.1186/s12889-016-3292-6

**Published:** 2016-07-22

**Authors:** Abdulbasit Musa, Nega Assefa, Fitsum Weldegebreal, Habtamu Mitiku, Zelalem Teklemariam

**Affiliations:** College of Health and Medical Sciences, Haramaya University, Harar, Ethiopia

**Keywords:** Kersa HDSS, Modern contraceptives, Ethiopia

## Abstract

**Background:**

Worldwide, every year 289,000 women die related to pregnancy and its complications. Nearly, all of these deaths occur in developing countries and more than half of this deaths occur in sub-Saharan Africa. Report suggested that using contraceptives can reduce this maternal mortality by 44 %. Even if, Ethiopia is one of the countries with highest maternal mortality, only 41 % of married women are using family planning. This analysis aimed at assessing factor associated with experience of contraceptive use before pregnancy among women who gave birth in Kersa Health and Demographic Surveillance System, Ethiopia.

**Methods:**

This study was part of data generated for Kersa Health and Demographic Surveillance System. Women who gave birth during October 2011 to September 2012 were asked whether they had used contraceptive before getting their last pregnancy. Data were collected by using Kersa Health and Demographic Surveillance System questionnaire. Both bi-variate and multivariate analysis were used to identify associated factors.

**Results:**

The proportion of modern contraceptive before pregnancy among the study participants was found to be 383 (40.9 %). The most commonly used modern contraceptives was Injectable contraceptive 270 (70.0 %) followed by oral contraceptives, 66 (17.23 %). Modern contraceptive use was negatively association with being Muslim (AOR = 0.2, 95 % CI = 0.05, 0.72) and being young mother (AOR = 0.44, 95 % CI = 0.22, 0.86). Rural town residence (AOR = 2.23, 95 % CI = 1.15, 4.35) was found to have positive association with utilization of modern contraceptives.

**Conclusions:**

Among women giving birth, only a minority had attempted to delay or prevent their recent birth by using contraception. Being young, being Muslim and living in rural area were significantly associated with low utilization of modern contraceptive. Increasing family planning education and involving religious leaders in family planning promotion would improve utilization of modern contraceptive use.

## Background

Worldwide 800 women die every day due to pregnancy or child birth related complications. Almost all maternal deaths (99 %) occur in developing countries and more than half of this deaths occur in sub-Saharan Africa [1].

Ethiopia, being one of the countries with highest maternal mortality in the world, is striving hard in reducing maternal mortality. The recent report on maternal mortality showed that, the country has reduced the ratio by 60 %. In the last 5 years, effort has been made in improving the health service in order to address the demand of pregnant and delivering women to avert morbidity and mortality associated with pregnancy and child birth. Even though there is substantial reduction in maternal mortality; still the Ethiopia is one of the countries with excess of maternal deaths [2, 3].

Family planning is one of the most effective strategies in reducing maternal and infant mortality. Family planning helps to avoid unwanted pregnancy and reduce risks of unsafe abortion. It can also prevent closely spaced and ill-timed pregnancies and births, which contribute to some of the world highest infant mortality rates. Infants of mothers who die as a result of giving birth also have a greater risk of death and poor health [3, 4]. Report suggested that only providing contraceptives for women can reduce maternal mortality by 44 % [5].

Cognizant of the importance of contraceptive in reducing maternal and infant mortality, Ethiopian Federal Ministry of Health is striving to achieve the planned goals of increasing Contraceptive prevalence rate to 66 % by the year 2015 [6]. According 2014 Mini Ethiopian Demographic and Health Survey Report (EDHS) report, Four in every ten currently married women (42 %) are using a method of contraception. Among the users of contraception reported by 2014 Mini EDHS, 95 % percents of women are using modern contraceptive, injectable being the most commonly used methods (77.5 %) of modern contraceptive [7, 8]. Hence; assessing factors that affect utilization of modern contraceptive is important to make intervention that speed up government effort in reducing maternal and child mortality.

## Methods

### Setting

**Fig. 1:**
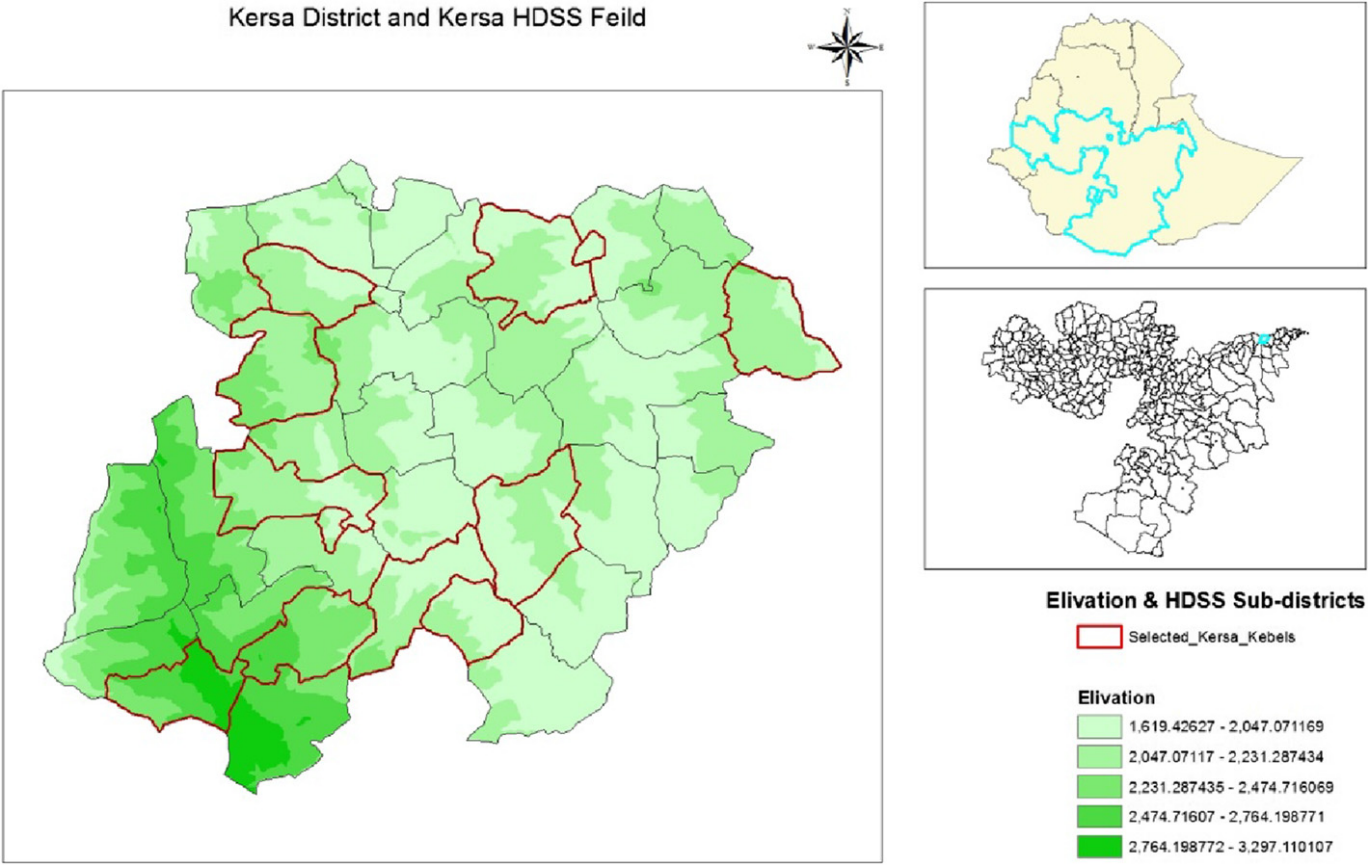
Kersa HDSS district and the 12 Sub-districts included in the surveillance process with altitude variation

The analysis used data from Kersa Health and Demographic Surveillance System (Kersa HDSS). Kersa HDSS is operated by Kersa Demographic Surveillance and Health Research Center (KDS-HRC) under Haramaya University. The field site is located between *41°40*^*”*^*0*^*’*^ and *41°57*^*”*^*30*^*’*^ easting and *09°15*^*”*^*15*^*’*^ and *09°29*^*”*^*15*^*’*^ northing. The surveillance system is located in the eastern Hararge zone, Oromiya Regional State, Eastern Ethiopia (Fig. 1). The district (Kersa District) has three climatic zones with the altitude ranging from 1600 to 3200 m above sea level. Based on figures published by the Central Statistical Agency in 2007, the district has an estimated total population of 170,816 of whom 86,134 (50.4 %) were males and 84,682 (49.4 %) were females; 11,387 or 6.67 % of its population are Rural town dwellers. The population in the district has crude birth rate of 37.2/1000, and Total fertility rate of 5.2. The district has seven health centers at different location of the district. In addition there are seven clinic/health posts and eight private pharmacies [9].

### Surveillance system, design, and population

Kersa HDSS was established with the aim of generating community based health and demographic events in the Eastern part of Ethiopia. The Kersa HDSS covers the whole population in 12 sub-districts of 38 sub-districts that are found in kersa, Eastern Ethiopia. At the start of the surveillance process, ethical clearance was secured from FDRE, National Ethical Clearance Board having the reference number of 3.10/313/03. Consent was obtained from the study participants after they were informed about the study, the objective, out come, benefits and risk associated with the study was given to the study participants.

It is an open cohort set up in 12 sub-districts of Kersa district. The site is principally rural including two small rural town (Kersa and Weter towns). The baseline census was done in 2007and since then a continuous population updated is being done twice every 6 months. During the updates, demographic and health events registered. Data is entered into the HRS-2 relational database. At the baseline 10,168 houses and 53,481 people were registered. The sex ratio and person per household was 1 and 5.1, respectively. At the end of 2013 the population becomes 63,000. Kersa HDSS is an INDEPTH member. INDEPTH is a network of Health and Demographic Surveillance Systems (www.indepth-network.org).

### Database and data extraction

The system uses HRS-2 database. The software is flexible and can export selected data to other software for analysis. This analysis used data collected from women who gave birth during October, 2011 to September 2012. The data was obtained from KDS-HRC through formal request. Open access is granted after the request is evaluated by kersa HDSS team. The detail data sharing policy of Kersa HDSS can be accessed at: [http://www.haramaya.edu.et/research/projects/kds-hrc/kds-hrc-project-data/].

### Data generation

Data were collected by using Kersa HDSS questionnaire. The questionnaire was adopted from EDHS and other relevant research report [2]. All mothers (937) residing in Kersa HDSS who gave birth their last child and identified for maternal health surveillance were asked about their modern contraceptive utilization before they conceived their last pregnancy. Modern contraceptive were defined as using any of the following contraceptives as defined by EDHS: Voluntary Surgical Contraception (VSC), the pill, Intra Uterine contraceptive Device (IUCD), injectables, implants, male and female condoms, lactational amenorrhea method, emergency contraception, and the standard days method [7].

### Variables

Age of the mother, residence, religion, maternal occupation, maternal education, partner’s education, and average monthly family income, access to media, number of children alive, and knowledge of contraceptive were used as explanatory variables. The outcome variable in this analysis is experience of modern contraceptive use before pregnancy. Modern Contraceptive use in this study refers to use of any modern contraceptive before they conceived their last child i.e. whether she had previous birth or not. Data were collected by personnel recruited for the surveillance purpose.

### Analysis

The results were presented in the form of tables, and text using frequencies and summary statistics such as mean, standard deviation and percentage to describe the study population in relation to relevant variables. Further, to identify factors associated with the outcome variable, logistic regression analysis was performed. Variables with *p* value ≤ 0.2 in the bi-variate analysis were considered for multivariate logistic regression model.

Variables having *p* value ≤ 0.05 in the multivariate analysis were taken as significant predictors. Crude and adjusted odds ratios with their 95 % confidence intervals were calculated. The Hosmer and Lemeshow goodness-of-fit test was used to assess whether the necessary assumptions for the application of multiple logistic regression were fulfilled and *p* value > 0.05 was considered a good fit.

## Result

### Characteristics of women

The mean age of the women was 28.6 (SD = 6.24) with more than half of the respondent, 489 (52.2 %), being in age group of 25–34.9 years. Majority, 890 (95.0 %) of the women resides in rural areas. Nine hundred and nineteen (98.1 %) of them were Muslim and 860 (91.8 %) were housewife by occupation. Seven hundred and fifty four of the women (80.5 %) and 563 (60.0 %) of their partner were illiterate. Regarding average monthly income of the participants, 364 (38.8 %) of the households have monthly income of less than 500 Ethiopian Birr. Four hundred and eighty one (51.3 %) of the participants have radio or Television in their house.

Concerning maternal health factors, most of the women 586 (54.0 %) had 1–4 children, only 57 (6.1 %) of the respondents reported to have had ever abortion, and 815 (87 %) of respondents heard about contraceptives, but only 383 (40.9 %) of respondent reported to have used contraceptives before being pregnant their last child. The most commonly used modern contraceptives was Injectable contraceptive 270 (70.0 %) followed by oral contraceptives 66 (17.23 %) (Table 1).

**Table 1:** Characteristics of women included in the analysis, Kersa HDSS, Ethiopia October 2012)

Variable		Number (%)	Percent
Age	<20 years	71	7.6
	20–24.9	248	26.5
	25–34.9	489	52.2
	35 and above	129	13.8
Residence	Rural Town	47	5.0
	Rural	890	95.0
Religion	Muslim	919	98.1
	Christian	18	1.9
Maternal Education	Educated	158	16.9
	Only read/Read and write	25	2.7
	Illiterate	754	80.5
Partners Education	Educated	271	28.9
	Only read/Read and write	82	8.8
	Illiterate	563	60.1
	Un known	21	2.2
Maternal occupation	House wife	860	91.8
	Student	29	3.1
	Farmer	15	1.6
	Other	33	3.5
Average family monthly income in Ethiopian Birr	<500	364	38.8
	500–999.9	404	43.1
	1000–1499.9	104	11.1
	1500 and above	65	6.9
Access to Media(TV/Radio)	Yes	481	51.3
	No	456	48.7
History of abortion	Yes	57	6.1
	No	867	92.5
	I don’t know	13	1.4
Numbers of child alive	No child	111	11.8
	One to four children	506	54.0
	Five and above children	320	34.2
Heard about contraceptive	Yes	815	87
	No	122	13
Used modern contraceptive	Yes	383	40.9
	No	554	59.1
Method used before pregnancy	Injectable contraceptive	270	70.5
	Oral contraceptive	66	17.23
	Implant	41	10.71
	IUCD	5	1.31
	VSC	1	0.26

### Factor associated with modern contraceptive use before pregnancy

On bivariate analysis, factors found to be significantly associated with use of modern contraceptives were; religion, age, place of residence, average monthly income in Ethiopian Birr and numbers of child alive. From these variables; Religion, age and place of residence were significantly and independently associated with utilization of modern contraceptives in multiple logistic regression analysis.

In this study, Muslim followers were 80 % (AOR = 0.2, 95 % CI = 0.05, 0.72) less likely to use modern contraceptive than Christians. In addition, women aged under 20 years were 66 % (AOR = 0.44, 95 % CI = 0.22, 0.86) less likely to use modern contraceptive compared to those above 35 years.

Regarding place of residence, women who reside in rural towns were 2.2 times (AOR = 2.23, 95 % CI = 1.15, 4.35) more likely to use modern contraceptive than those who resides in rural (Table 2).

**Table 2:** Factors associated with contraceptive use before pregnancy among women who gave birth during 2011–12 in Kersa HDSS, Ethiopia

Variable	Use of FP before pregnancy		COR(95 % CI), *P*-value		AOR(95 % CI), *P*-Value	
	Yes	No	COR(95 % CI),	*P*-value	AOR(95 % CI)	*P*-Value
	Number (%)	Number (%)				
Religion of the mother						
Muslim	368(40.0)	551(60.0)	0.13(0.04, 0.47)	0.002	0.2(0.05, 0.72)*	0.014
Christian	15(83.3)	3(16.7)	1			
Age of the mother in year						
Less than 20	16(22.5)	55(77.5)	0.47(0.24,0.91)	0.026	0.44(0.22,0.86)*	0.017
20–24.9	97(39.1)	151(60.9)	0.92(0.60,1.41)	0.705	0.91(0.58,1.41)	0.661
25–34.9	219(44.8)	270(55.2)	1.18(0.80,1.74)	0.416	1.14(0.76,1.70)	0.528
35 and above	51(39.5)	78(60.5)	1		1	
Residence						
Urban	30(63.8)	17(36.2)	2.69(1.46,4.94)	0.002	2.23(1.15,4.35)*	0.018
Rural	353(39.7)	537(60.3)	1		1	
Average family monthly income in Eth Birr						
< 500	161(44.2)	203(55.8)	0.87(0.51,1.48)	0.605	0.97(0.56,1.68)	0.906
500–999	139(34.4)	265(65.6)	0.58(0.34,0.98)	0.040	0.64(0.37,1.11)	0.116
1000–1499	52(50.0)	52(50.0)	1.02(0.59,2.04)	0.770	1.21(0.64,2.31)	0.553
1500 and above	31(47.7)	34(52.3)	1		1	
No of child alive						
No alive child	33(29.7)	78(70.3)	1			
1–4 children alive	208(41.1)	298(58.9)	1.65(1.06,2.57)	0.027	1.25(0.74,2.12)	0.245
5 and above children	142(44.4)	178(55.6)	1.89(1.19,2.10)	0.007	1.42(0.79,2.54	0.117

* Significant at *p*-value of ≤0.05, *AOR* adjusted odds ratio, *COR* crude odds ratio, *CI* confidence interval

Variables used in multivariate model; religion of the mother, age of the mother, place of residence, average family monthly income in Ethiopian Birr and numbers of child alive

## Discussion

This analysis identifies factor associated with experience of modern contraceptive use among women who have registry of pregnancy outcome in Kersa HDSS. The proportions of women who used modern contraceptive were found to be 41 %. These are women who successfully or unsuccessfully attempted to delay or prevent their most recent pregnancy.

Majority of the respondents (70 %) reported to use injectable contraceptive. This finding is consistent with other studies done in Ethiopia [10–16]. This choice could be due to its convenience of not being taken on daily basis as evidenced by another research [17].

In this study, Muslim women were 80 % less likely to use modern contraceptives than Christians. This finding is in agreement with the study conducted in Ghana [18]. This might be due to the wide believe in the Muslim community that consider family planning prohibited in the holly book as indicated in another report [19].

Women aged less than 20 years were 66 % less likely to use modern contraceptive compared to those above 35 years. This finding is consistent with study from India [20]. This might be due to the fact that younger women who are married are more likely to want pregnancy to have their first child. In addition, those who want to delay pregnancy might relay on traditional methods than modern contraceptive as they fear perceived infertility as reported by another article [21].

Women who were residing in rural town were 2.2 times more likely to use modern contraceptives than those who were rural resident. This finding is consistent with the studies from Butajira [15], Dambia [14], and national survey conducted in 2014 [13]. This might be due to those women living in the rural town have better access to health information from different sources including media.

A limitation of this analysis is that important variables that may affect utilization of modern contraceptive such as husband approval of contraceptive use, couples discussion, and support from partner which has association in another studies were missed from the system [15, 22]. The main reason for this is, the data is extracted from existing HDSS database and not primarily done for family planning study purpose. In addition, variable like family income who had association in another study [23] was not found to have significant association with modern contraceptive utilization in this study. This might be due to the fact that contraceptive service in Ethiopia is free of any cost, so that income should not be a barrier for the service. Similarly, factors like access to media, number of living children, and maternal education which had association in other studies [24, 25] failed to show any association in this study. So, the authors believe that further research should be conducted to answer such disparity. The reader should also take in consideration that since fewer than 2 % of the respondents were non-Muslim, the association between religion and contraceptive use should not be generalizable to other areas.

Regardless of these limitations, this study was the first to be conducted in this geographic area and its finding can provide valid information regarding modern contraceptive utilization having relatively larger participants.

## Conclusions

Among women giving birth, only a minority had attempted to delay or prevent their recent birth by using modern contraception. Being young, being Muslim and living in rural area were significantly associated with low utilization of modern contraceptive. Providing family planning education for the communities would enhance its uptake. Involving religious leaders in family planning promotion is recommended in improving utilization of modern contraceptive.

## Abbreviation

EDHS, Ethiopian Demographic and Health Survey; HDSS, Health and Demographic Surveillance System; IUCD, Intra Uterine Contraceptive Device; KDS-HRC, Kersa Demographic Surveillance and Health Research Center; VSC, Voluntary Surgical Contraception
